# Effect of the Profit and Teaching Status of Hospitals on the Patterns and Outcomes of Abdominal Aorta and Inferior Vena Cava Injuries after Severe Abdominal Trauma

**DOI:** 10.1155/2023/5616007

**Published:** 2023-08-11

**Authors:** Nasser A. N. Alzerwi

**Affiliations:** Department of Surgery, College of Medicine, Majmaah University, Ministry of Education, P. O. Box 66, Al-Majmaah 11952, Riyadh, Saudi Arabia

## Abstract

**Background:**

The inferior vena cava (IVC) and the abdominal aorta (AA) are two important blood vessels located in the abdomen. The outcomes of such injuries rely heavily on the experience, expertise, and resources available at the hospital where the patient is treated. However, our current understanding of the potential impact of the hospital profit and teaching status on surgical outcomes in the context of traumatic injuries to the IVC and AA remains limited, making it important to investigate the potential association between these hospital characteristics and patient outcomes to enhance the quality of care and optimize treatment strategies.

**Objective:**

This study aimed to compare demographics, trauma characteristics, and outcomes between nonprofit status (NPSH) and for-profit hospital status (FPSH), as well as among community hospitals (CHs), nonteaching hospitals (NTHs), and university hospitals (UHs), in patients with severe abdominal trauma and abdominal aorta injury (AAI), inferior vena cava injury (IVCI), and both (AAI + IVCI).

**Methods:**

Demographics, trauma, and outcome measures associated with AAI, IVCI, and AAI + IVCI were compared between the different profit and teaching status groups using NTDB. Multivariate regression was used to identify independent factors associated with death under care (DUC).

**Results:**

In the 2017 NTDB-RDS, 1,479 patients met the inclusion criteria, resulting in an overall incidence of 0.17% for AAI, IVCI, and AAI + IVCI after severe abdominal trauma. More patients died under care in the FPSH group than in the NPSH group (nonprofit vs. for-profit: 60.3% vs. 47.2%; *P* < 0.001). The results indicated that FPSH independently affected DUC. NTH had no significant effect on DUC; although the in-hospital complication rate varied with NTH, no independent association was observed.

**Conclusions:**

The study findings demonstrated that in patients with severe abdominal trauma, including injuries to AAI, IVCI, or both (AAI + IVCI), the profit status of hospitals, rather than the teaching status, had a substantial influence on DUC. Future studies should examine differences in the volume of cases and levels of trauma centers to better understand how to improve patient outcomes in FPSH.

## 1. Introduction

The abdominal aorta (AA), the largest artery in the abdomen, carries oxygen-rich blood from the heart to the lower body. The inferior vena cava (IVC) is the largest vein in the abdomen and carries deoxygenated blood back to the right atrium [1]. AA and IVC constitute the major blood vessels in the abdomen and are essential for proper body function. As expected, trauma to these vessels can be life-threatening and requires immediate medical attention. IVC injury (IVCI) remains a severe problem in the operating room because it generates significant blood loss and has a high mortality rate. Abdominal aorta injury (AAI) is also potentially fatal, accounting for 0.1% of all trauma hospitalizations [2].

Despite breakthroughs in diagnosis and management, these traumas are associated with substantial morbidity and fatality rates [3], and because AAI and IVCI are rare, their clinical features are not well understood. Furthermore, the reported prevalence and outcomes vary among studies due to differences in expertise and facilities available at hospitals. The lack of uniformity in the number of cases across different study centers makes it challenging to accurately determine mortality rates adjusted for the number of patients with these conditions and to analyze other related factors [1]. The healthcare community debates whether the outcomes of medical care vary depending on the hospital's teaching status (TS) [4–7]. Teaching hospitals are commonly considered to provide high-quality treatment, with public opinion generally favoring them. Studies suggest that the major TS is associated with lower mortality rates for common conditions than non-TS hospitals [5, 6]. Similarly, there has been significant interest in examining the effect of for-profit hospitals (FHSs) on care and outcomes [8, 9]. To properly handle IVCI and AAI, hospitals require specialist resources and facilities, given the complexity of these injuries. A robust postoperative care system with access to critical care and expert nursing is essential to manage potential difficulties and ensure optimal recovery. Given such constraints, the profitability and teaching status of hospitals can become relevant factors [4, 6–9]. As nonprofits, some hospitals may be better positioned to devote resources to essential care services based on their distinct focus and goals. Teaching hospitals often attract highly qualified medical personnel by focusing on training and research. They also provide specialized training programs that add to the expertise available for managing challenging IVCI and AAI cases. Investigating how the hospital profit status and teaching status impact patient outcomes is crucial to ensure that hospitals have the knowledge, effective trauma systems, and infrastructure necessary to deliver improved results for IVCI and AAI patients [4].

The National Trauma Data Bank (NTDB) is a large database that collects and houses data on trauma patients from hospitals in the United States. It aims to improve the quality of care for trauma patients by providing a centralized data source for researchers, healthcare providers, and policymakers. NTDB is updated annually and contains data from more than 1,000 hospitals in the United States. It is a valuable resource for anyone interested in understanding the epidemiology and treatment of traumatic injuries, and its data have contributed to significant advances in trauma care over the past several decades. However, to our knowledge, no NTDB study has examined the effect of THS and FHS on the care and outcomes of patients with severe abdominal trauma, IVCI, and AAI.

This study aimed to examine the pattern and outcome of AAI and IVCI in hospitals with different teaching statuses (TSs) and profit statuses (PSs). Efforts were made to identify the factors independently associated with death under care (DUC) in patients with severe abdominal trauma, AAI, and IVCI.

## 2. Methodology

We retrospectively analyzed secondary data from the 2017 National Trauma Data Bank Research Dataset (NTDB-RDS). Patients with AAI and/or IVCI were identified using the International Classification of Diseases ICD-10-CM diagnosis code (S35.0 or S35.1) and were stratified according to the type of injury (AAI, IVCI, and AAI + IVCI), PS (for-profit status hospital (FPSH) and nonprofit hospital (NPSH)), and TS (community hospital (CH), nonteaching hospital (NTH), and university hospitals (UH)). Only patients with severe abdominal trauma (abbreviated injury scale (AIS) grade ≥ 3) were included. DUC included all in-hospital mortality cases. Demographic and trauma-related characteristics included age, sex, race, ISS, SBP, Glasgow Coma Scale (GCS) score, and AIS score. Outcome measures included DUC, sepsis, and overall complication rates. Demographic characteristics and outcome measures were collected and compared between the groups.

We employed STATA 16 statistical software to analyze the data, and statistical significance was set at *P* < 0.05. We used the Mann–Whitney U and Kruskal–Wallis tests for continuous variables after confirming nonnormal distribution, chi-square for categorical variables, and logistic regression for ORs. For DUC and in-hospital complications, we calculated the odds ratios (ORs) by age, ISS, SBP, GCS, AAI/IVCI, TS, and NPSH.

## 3. Results

### 3.1. General Characteristics of Patients

In the 2017 NTDB-RDS, 1,479 patients met the inclusion criteria, resulting in an overall incidence of 0.17% for AA and IVC (Tables S1 and S2). The median age of the study participants was 33.0 years, with 52.1% in the age group 21–44. Most patients were male (77.6%), and 49.6% were white. Of the patients, 70.7% had ISS scores of >25. Blunt and penetrating trauma (both individually close to 50%) accounted for most of the injuries. The most common injury mechanism was firearms (43.5%), followed by motor vehicle accidents (MVAs) (39.7%), falls (5.7%), stabbing (4.6%), and other causes (6.4%). In most cases, the intent was unintentional (52.2%), followed by assault (40.8%), self-infliction (3.7%), and others (3.2%).

**Figure 1:**
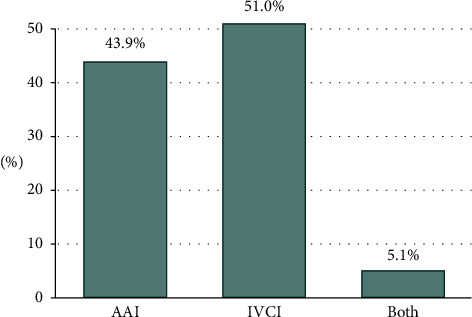
Distribution of abdominal aorta injury (AAI), inferior vena cava injury (IVCI), and both types of injuries in this study population.

**Figure 2:**
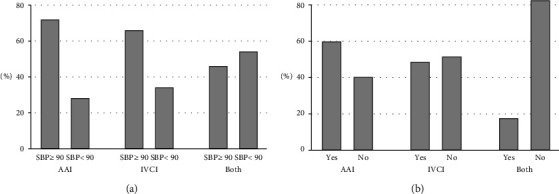
Abdominal aorta injury (AAI)/inferior vena cava injury (IVCI). (a) Percentage of patients with systolic blood pressure (SBP) ≤ 90 mmHg. (b) Death under care (DUC) (%).

The median LOS (IQR) (hospital) was 5.0 (1.0, 15.0) days. Approximately 43.9% of the patients had AAI, 51.0% had IVCI, and 5.1% had both (Figure 1). A total of 48.1% of the patients died during care, and the complication rate was 34.0%. The prevalence of sepsis was 4.7%. Figures 2(a) and 2(b) show the hemodynamic status and DUC in the AAI/IVCI group. The results clearly demonstrated that patients with both AAI and IVCI had the highest mortality rates. Furthermore, the IVCI group exhibited a significantly higher DUC than the AAI group, indicating that IVCI is associated with a greater risk of mortality than AAI in this patient population. Since this study aimed to investigate the impact of the profit status (PS) and teaching status (TS) on hospital outcomes, the subsequent analysis focused on examining the effects of AAI, IVCI, and AAI + IVCI in different subgroups of hospitals based on their PS and TS.

### 3.2. Nonprofit vs. Profit Status

Most of the patients were treated in nonprofit hospitals (Table 1). However, there was no discernible age difference between patients admitted to nonprofit or for-profit institutions (nonprofit vs. for-profit: 37.5 y (26.0, 51.5) vs. 33.0 y (23.0, 49.5); *P*=0.064) nor was there any significant difference in the percentage of patients with an ISS > 25 (nonprofit vs. for-profit: 68.5% vs. 71.3%; *P*=0.673). The type of injury and trauma mechanism were not significantly different between the two groups, with firearms being the most common mechanism and penetrating and blunt trauma, accounting for more than 99% of cases. The racial makeup of the patients did not differ significantly between the NPSH and FPSH groups. However, there was a significant difference in the number of hospital beds, with 44.7 percent of the nonprofit hospitals having more than 600 beds compared to 10.3 percent of FPSH (*P* < 0.001). Trauma-level centers were also significantly different, with a higher proportion of level 1 trauma centers in nonprofit hospitals (nonprofit vs. for-profit: 39.1% vs. 78.4%; *P* < 0.001). Although the type of injury and trauma mechanism did not differ between the two groups, there was a slight difference in the pattern of the type of intent, which was statistically significant.

There were no differences in vital signs between the groups with SBP < 90 mmHg (nonprofit vs. for-profit: 37.0% vs. 32.1%; *P*=0.231; Table 2) and GCS ≤ 8 (nonprofit vs. for-profit: 47.3% vs. 39.5%; *P*=0.070). The length of hospital stay was longer for patients treated in nonprofit hospitals than for those treated in FPSH (nonprofit vs. for-profit: 2.0 d (1.0, 11.0) vs. 5.0 d (1.0, 15.0); *P*=0.006). More patients died under the care of for-profit hospitals than of nonprofit hospitals (nonprofit vs. for-profit: 60.3% vs. 47.2%; *P* < 0.001). The incidence of complications during hospitalization was similar between nonprofit and for-profit hospitals (nonprofit vs. for-profit: 32.2% vs. 34.3%; *P*=0.61). The prevalence of sepsis was slightly higher in FPSH (3.4%) than in nonprofit hospitals (1.9%), but the difference was not statistically significant (*P*=0.224).

### 3.3. Teaching Status

Most of the patients (>50%) received treatment in the UH. The age of patients admitted to CH, NTH, or UH was not significantly different (CH vs. NTH vs. UH: 32.0 (24.0, 51.0) vs. 33.5 (23.0, 51.0) vs. 33.0 (23.0, 49.0); *P*=0.924; Table 2). Similarly, there were no significant differences in the percentage of patients with ISS > 25 (CH vs. NTH vs. UH: 69.7% vs. 65.9% vs. 72.4%; *P*=0.924). The type of injury and trauma mechanism was also not significantly different between the hospital types, with firearms being the most common mechanism and penetrating and blunt trauma, accounting for over 99% of the cases. The racial makeup of the patients was also similar between nonprofits and FPH. However, the number of hospital beds differed significantly, with >600 beds in 27.7%, 26.2%, and 49.7% of CH, NTH, and UH, respectively (*P* < 0.001). Trauma-level centers were also significantly different, with 49.8%, 13.8%, and 94.6% of level 1 trauma centers in CH, NTH, and UH, respectively (*P* < 0.001). However, the type, mechanism, and intent of the injury did not differ significantly between the three hospital groups.

### 3.4. Factors Affecting DUC

Multivariate analysis was performed to investigate whether TS or PS affected DUC and in-hospital complications (Tables 3 and S3). The analysis considered common predictors such as age, systolic blood pressure (SBP), GCS, injury severity score (ISS), AAI/IVCI, TS, and PS. The results indicated that PS was an independent risk factor for DUC, in addition to ISS, SBP, GCS, and AAI/IVCI. The analysis also yielded a receiver operating characteristic (ROC) curve with an area under the curve (AUC) of approximately 0.9, suggesting that the model had good predictive power.

Interestingly, the subgroup analysis showed substantial variations in complication rates, number of beds, and level 1 trauma centers in CH, NTH, and UH. As a result, we used multivariate analysis to identify predictors of complications by including common characteristics, such as age, ISS, SBP, GCS, PS, and TS. Unfortunately, the quality of the regression model was inadequate (ROC = 0.55); therefore, these data were not included in this study. It is worth noting that the model did not find an independent relationship between the complication rate and TS or PS (Table S3).

## 4. Discussion

Traumatic abdominal vascular injuries are often serious, and trauma management can be challenging due to high rates of mortality and complications [10]. This study sought to explain DUC in patients with these injuries and examine whether TS and PS can influence the mortality rate. It is noteworthy that our study found that PS and type of vascular injury (AAI, ICVI, or both) affected the mortality rate, in addition to commonly expected variables such as age, injury severity, coma status, and hemodynamic stability.

Our study did not find evidence of racial bias or preference in patients who underwent AAI/IVCI treatment at FPSH or NPSH. There were also no significant differences in age, trauma, intent, mechanism, or type between the PS types. However, there were some notable differences, such as the proportion of hospitals with 600+ beds being higher in NPSH, with level 1 trauma centers almost doubling. Patients in the NPSH group also had longer stays than those in the FPSH group. The frequency of DUC was significantly higher in the FPSH group although there were no significant differences in the SBP, GCS, and ISS scores. When using multivariate logistic regression to identify predictors of DUC, the PS status was found to be independently associated with the risk of DUC, as well as common factors such as age, ISS, SBP, and GCS. Patients with injuries to both AAI and IVCI had almost double the risk of DUC, and the combined AAI + IVCI injury increased the risk by more than six times compared with AAI and three times that of IVCI.

Interestingly, TS did not have any significant effect on DUC. The TS subgroup analysis did not reveal any differences in DUC; therefore, the results of the multivariate analysis were in line with expectations. Interestingly, the subgroup analysis revealed significant differences in the rates of complications, number of beds, and level 1 trauma centers among CH, NTH, and UH. Therefore, we performed multivariate analysis to identify predictors of complications, including common factors such as age, ISS, SBP, GCS, PS, and TS. Unfortunately, the quality of the regression model was poor (ROC ∼ 0.55); therefore, these findings were not included in this study. It should be noted that the model did not demonstrate any independent association between the complication rate and TS or PS (Table S2). In a recent study, Elkbuli et al. examined popliteal vascular injury in different TS subgroups. These authors found that patients with popliteal vascular injuries treated at a CH had a mortality risk 12.3 times higher than those treated at another hospital with similar injuries. Similarly, patients treated at a university hospital had a mortality risk 5.6 times higher than those treated at a nonteaching hospital. However, after accounting for confounding factors, these differences were no longer statistically significant [11]. Our study also found differences in the complication rates between these types but no significant differences in terms of DUC.

In addition, our study found that AAI/IVCI had a high DUC (48.1%) and that there were substantial disparities among AAI (DUC = 40%), IVCI (DUC = 50%), and combined AAI + IVCI (DUC = 80%). Blunt AAI has historically been associated with significant morbidity and mortality [12] although mortality in patients who survive presentation tends to decrease [2, 13]. Branco et al. conducted a study on AAI caused by BT or PT and discovered that the mortality rate following AAI was 30.4% in 2002 and jumped to 66.0% in 2014 for PT. However, for BT, the mortality rate after AAI decreased from 58.3% in 2002 to 26.2% in 2014 [14]. de Mestral et al. conducted a study using NTDB to investigate the pattern, treatment, and in-hospital outcomes of patients with blunt AAI. Their findings indicated that the mean ISS was 35 ± 14, most patients experienced injuries from motor vehicle accidents, and the overall mortality rate was 29% [15]. Another notable study found that among 392,315 blunt trauma patients, 113 (0.03%) had AAI. The median age of the patients was 38 years, and the ISS was 34. The predominant cause of injury was motor vehicle accidents (60%), and hypotension was observed in 47% of cases, along with spinal fractures (44%) and pneumothorax or hemothorax (42%) as significantly related complaints. Most deaths (68%) were due to hemorrhage or cardiac arrest and occurred during the first 24 hours [16]. The first NTDB study on AAI, involving 3,114 patients, found an overall incidence of 0.3%. Compared to matched controls, AAI resulted in a higher mortality rate (55% vs. 15%) and independently contributed to mortality. This study was the first to define the incidence of BAI using NTDB [17]. Shalhub et al. discovered that blunting an AAI is a rare injury, with fewer than 200 cases reported at the time. These authors found a median ISS of 45 and 39%, respectively, for hypotensive individuals. The overall mortality rate was 32% [18, 19].

In our study, which focused only on patients with severe abdominal trauma, the incidence rate of AAI/IVCI was 0.17%. This low number can be attributed to the study's focus on only severe cases. In our study, the mortality rate was approximately 50%, reflecting the severity of these injuries and highlighting the importance of appropriate trauma treatment at the emergency response and hospital level. This study examined the demographics, injury patterns, mortality, and complication rates in hospitals with different PS and TS statuses. Our results indicate that despite breakthroughs in resuscitation and critical care, they remain extremely lethal. Few studies have examined the impact of PS and TS, which are rare in trauma management. When looking at the mortality rates for specific categories of injuries, it was found that individuals with AAI performed better than those who had IVCI or a combination of AAI and IVCI. However, unlike Elkbuli et al., who found that this pattern depends on TS [11], we did not observe any significant variation in mortality associated with TS. Our results support those of a systematic review of the impact of TS, revealing that TS does not significantly influence clinical outcomes. However, variations in individual diseases cannot be ruled out although they are unlikely to be significant [4].

However, we observed the lowest complications in the UH group, supporting the hypothesis of Chiu et al. that UHs are more experienced in managing vascular injuries because they see a higher volume of these types of patients. FPSH is associated with higher inpatient mortality, length of stay, and hospital charges than their nonprofit counterparts [9]. However, we did not find any proof that FPSH preferentially serves healthier patients, delivers less evidence-based treatment, reduces hospital stays, or has patients with worse acute outcomes than nonprofit facilities [8]. In our study, the volume of cases handled in the UH was more significant than that of the total number of cases handled by the CH and NTH. The same reasoning may also explain, at least in part, the lower DUC in PS. It is reasonable to assume that they handled almost ten times more cases in 2017, demonstrating their greater experience in managing such cases. Another factor could be the level of trauma since almost 80% of the trauma centers in the NPS were level 1 compared to only 40% in the FPSH.

### 4.1. Limitations

This study has certain limitations, primarily stemming from the utilization of a retrospective database. In addition, reliance on user-contributed data within NTDB introduces the possibility of injury misclassification, complications, inconsistent reporting, and substantial variability. These factors may have influenced the DUC rates reported in this study. Furthermore, it should be noted that the NTDB dataset does not encompass improved prehospital treatment or the transfer of critically wounded patients from the accident scene to a trauma center, which could potentially account for the increased fatality rate. Another constraint lies in the incompleteness and missing data points within NTDB, limiting researchers' ability to draw comprehensive conclusions or make inferences regarding certain aspects of patient injury and care. The generalizability of the findings to broader populations is also subject to caution, as NTDB primarily includes trauma centers within the United States, potentially limiting its applicability to other countries or individuals not seeking care at trauma centers. Furthermore, inconsistencies in data collection and reporting across institutions can introduce bias, which affects the reliability and validity of the data. While NTDB provides information on patient injury and outcomes, it may not offer detailed clinical insights into specific treatments, interventions, or the influence of comorbidities on the observed results. Another important limitation of this study is the lack of longitudinal data within NTDB. Therefore, relying solely on available NTDB data may not fully capture the long-term implications and outcomes related to the injuries under investigation.

### 4.2. Implications

The results of this study suggest that the hospital profit status significantly affects mortality rates in patients with severe abdominal injuries involving damage to the abdominal aorta and inferior vena cava. Policymakers should examine the reasons for this relationship and find ways to improve outcomes in for-profit hospitals. Healthcare professionals should be educated to properly identify and manage patients with abdominal trauma to improve their outcomes.

Further research on abdominal aortic and vena cava injuries should focus on several key areas to improve patient care. First, differences in trauma center levels and case volumes, especially in for-profit hospitals, should be explored. Understanding how these factors affect patient outcomes can inform resource allocation, trauma center designation, and case management strategies to optimize care. Including diverse patient populations from various healthcare systems will also provide a broader understanding of the impact of outcomes in these cases. This will ensure that any intervention developed based on the findings can benefit a wider range of patients with these abdominal injuries.

## 5. Conclusions

This study examined the differences between hospital types (profit and nonprofit) and TS (community, nonteaching, and university) with respect to the pattern of intent and mechanisms of trauma, response time, LOS, vital signs, and death under care. The national incidence of vascular injuries was 0.17% among all injured patients in NTDB in 2017. NFH had lower DUC. The reason for this is uncertain; however, it could be related to better perioperative care, which allows more critically wounded patients to arrive alive at the hospital due to the crucial amount of expertise and facilities available at the hospital. This study answers several unsettled questions about the hospital status and trauma outcomes by analyzing race, age, vital signs, and mortality and encourages future studies to explore the impact of case volume and trauma center level on achieving better outcomes. This study highlights the need to improve outcomes in patients with abdominal trauma, especially in for-profit hospitals. Additional research on trauma center factors and diverse patient populations can help develop evidence-based guidelines to manage aortic and vena cava injuries and reduce mortality rates. Policy changes and education for healthcare professionals may also help to address the disparities identified in this study.

## Figures and Tables

**Table 1:** Demographic characteristics by the type of trauma center, profit status, and hospital teaching status.

Variables	PS			TS			
	For-profit	Nonprofit	*P* values	Community hospital (CH)	Nonteaching hospital (NTH)	University hospital (UH)	*P* values
	(*N* = 146)	(*N* = 1306)		(*N* = 429)	(*N* = 126)	(*N* = 897)	
Age group			0.110				0.074
<16	4 (2.7%)	59 (4.6%)		14 (3.3%)	1 (0.8%)	48 (5.4%)	
16–20	7 (4.8%)	141 (10.9%)		49 (11.5%)	15 (12.0%)	84 (9.5%)	
21–44	81 (55.5%)	670 (52.0%)		214 (50.4%)	71 (56.8%)	466 (52.7%)	
45–64	37 (25.3%)	265 (20.6%)		85 (20.0%)	26 (20.8%)	191 (21.6%)	
65+	17 (11.6%)	153 (11.9%)		63 (14.8%)	12 (9.6%)	95 (10.7%)	
Sex			0.466				0.878
Men	117 (80.1%)	1012 (77.5%)		336 (78.3%)	96 (76.2%)	697 (77.7%)	
Women	29 (19.9%)	294 (22.5%)		93 (21.7%)	30 (23.8%)	200 (22.3%)	
Race			0.075				0.061
White	62 (42.5%)	656 (50.2%)		198 (46.2%)	55 (43.7%)	465 (51.8%)	
Injury type			0.687				0.369
Blunt	71 (48.6%)	677 (52.1%)		233 (54.4%)	63 (50.8%)	452 (50.6%)	
Penetrating	75 (51.4%)	622 (47.8%)		194 (45.3%)	61 (49.2%)	442 (49.4%)	
Others	0 (0.0%)	1 (0.1%)		1 (0.2%)	0 (0.0%)	0 (0.0%)	
Mechanism			0.462				0.450
MVA	51 (35.2%)	515 (39.8%)		174 (41.0%)	45 (36.0%)	347 (39.0%)	
Firearms	65 (44.8%)	565 (43.7%)		172 (40.6%)	55 (44.0%)	403 (45.3%)	
Fall	11 (7.6%)	72 (5.6%)		30 (7.1%)	6 (4.8%)	47 (5.3%)	
Cut/peirce	10 (6.9%)	57 (4.4%)		22 (5.2%)	6 (4.8%)	39 (4.4%)	
Others	8 (5.5%)	84 (6.5%)		26 (6.1%)	13 (10.4%)	53 (6.0%)	
Intent			0.021				0.198
Unintentional	72 (49.3%)	682 (52.2%)		237 (55.2%)	68 (54.0%)	449 (50.1%)	
Self-inflicted	7 (4.8%)	48 (3.7%)		9 (2.1%)	4 (3.2%)	42 (4.7%)	
Assault	56 (38.4%)	539 (41.3%)		169 (39.4%)	48 (38.1%)	378 (42.1%)	
Others	11 (7.5%)	37 (2.8%)		14 (3.3%)	6 (4.8%)	28 (3.1%)	
Trauma center level			<0.001				<0.001
Level I	36 (39.1%)	790 (78.7%)		163 (49.8%)	11 (13.8%)	652 (94.6%)	
Level II	56 (60.9%)	181 (18.0%)		146 (44.6%)	58 (72.5%)	33 (4.8%)	
Level III	0 (0.0%)	33 (3.3%)		18 (5.5%)	11 (13.8%)	4 (0.6%)	
Beds			<0.001				<0.001
≤ 200	16 (11.0%)	89 (6.8%)		43 (10.0%)	16 (12.7%)	46 (5.1%)	
201–400	53 (36.3%)	257 (19.7%)		155 (36.1%)	46 (36.5%)	109 (12.2%)	
401–600	62 (42.5%)	377 (28.9%)		112 (26.1%)	31 (24.6%)	296 (33.0%)	
>600	15 (10.3%)	583 (44.6%)		119 (27.7%)	33 (26.2%)	446 (49.7%)	

**Table 2:** Outcome measures by the type of injury and the hospital teaching status.

Variables	PS			TS			
	For-profit	Nonprofit	*P* values	Community	Nonteaching	University	*P* values
SBP			0.231				0.366
SBP ≥ 90	92 (63.0%)	887 (67.9%)		299 (69.7%)	80 (63.5%)	600 (66.9%)	
SBP < 90	54 (37.0%)	419 (32.1%)		130 (30.3%)	46 (36.5%)	297 (33.1%)	
GCS			0.070				0.754
GCS > 8	77 (52.7%)	790 (60.5%)		253 (59.0%)	79 (62.7%)	535 (59.6%)	
GCS ≤ 8	69 (47.3%)	516 (39.5%)		176 (41.0%)	47 (37.3%)	362 (40.4%)	
ISS			0.673				0.401
1–8	0 (0.0%)	1 (0.1%)		0 (0.0%)	0 (0.0%)	1 (0.1%)	
9–15	9 (6.2%)	54 (4.1%)		25 (5.8%)	6 (4.8%)	32 (3.6%)	
16–24	37 (25.3%)	320 (24.5%)		105 (24.5%)	37 (29.4%)	215 (24.0%)	
>25	100 (68.5%)	931 (71.3%)		299 (69.7%)	83 (65.9%)	649 (72.4%)	
AAI/IVCI			0.210				0.089
AAI	67 (45.9%)	572 (43.8%)		211 (49.2%)	53 (42.1%)	375 (41.8%)	
IVCI	76 (52.1%)	663 (50.8%)		196 (45.7%)	69 (54.8%)	474 (52.8%)	
Both	3 (2.1%)	71 (5.4%)		22 (5.1%)	4 (3.2%)	48 (5.4%)	
Associated injury regions							
(1) Head/neck			0.985				0.369
	9 (6.2%)	80 (6.1%)		31 (7.2%)	5 (4.0%)	53 (5.9%)	
(2) Face			0.636				0.538
	0 (0.0%)	2 (0.2%)		0 (0.0%)	0 (0.0%)	2 (0.2%)	
(3) Chest			0.508				0.974
	78 (53.4%)	660 (50.5%)		220 (51.3%)	64 (50.8%)	454 (50.6%)	
(4) Abdomen			—				—
	146 (100.0%)	1306 (100.0%)		429 (100.0%)	126 (100.0%)	897 (100.0%)	
(5) Extremities			0.118				0.591
	20 (13.7%)	248 (19.0%)		81 (18.9%)	19 (15.1%)	168 (18.7%)	
(6) External			0.179				0.595
	0 (0.0%)	16 (1.2%)		3 (0.7%)	2 (1.6%)	11 (1.2%)	
Complications			0.610				0.005
Discharged to facility			0.375				0.151
	0 (0.0%)	7 (0.5%)		1 (0.2%)	2 (1.6%)	4 (0.4%)	
Died under care			0.002				0.364
	88 (60.3%)	612 (46.9%)		219 (51.0%)	58 (46.0%)	423 (47.2%)	
Complications							
DVT			0.437				0.870
	4 (2.7%)	53 (4.1%)		16 (3.7%)	6 (4.8%)	35 (3.9%)	
Cardiac arrest			0.194				0.002
	23 (15.8%)	157 (12.0%)		56 (13.1%)	3 (2.4%)	121 (13.5%)	
Intubation			0.877				0.256
	4 (2.7%)	33 (2.5%)		10 (2.3%)	6 (4.8%)	21 (2.3%)	
Kidney			0.717				0.362
	7 (4.8%)	72 (5.5%)		20 (4.7%)	10 (7.9%)	49 (5.5%)	
Sepsis			0.224				0.923
	5 (3.4%)	25 (1.9%)		8 (1.9%)	3 (2.4%)	19 (2.1%)	

AAI: abdominal aorta injury, GCS: Glasgow Coma Scale, GSW: gunshot wound, ICU: intensive care unit, ISS: injury severity score, IVCI: inferior vena cava injury, LOS: length of stay, MVA: motor vehicle accidents, and SBP: systolic blood pressure.

**Table 3:** Odds ratio of DUC according to the type of injury, teaching status, profit status, and other vital parameters.

Variables	OR (95% CI)	*P* values
Age	1.013 (1.005–1.021)	0.001
ISS	1.035 (1.023–1.046)	<0.001
SBP	2.902 (2.144–3.928)	0.001
GCS	10.84 (8.065–14.569)	0.001
AAI/IVCI		
AAI	1	
IVCI	2.138 (1.597–2.863)	0.001
AAI/IVCI	6.44 (3.092–13.41)	0.001
Teaching status (TS)		
CH	1	
NTH	0.783 (0.466–1.315)	0.355
UH	0.792 (0.58–1.081)	0.141
Profit status (NPSH)	0.574 (0.361–0.913)	0.019

AAI: abdominal aorta injury, GCS: Glasgow Coma Scale, ISS: injury severity score, IVCI: inferior vena cava injury, MVA: motor vehicle accidents, and SBP: systolic blood pressure.

## Data Availability

The data used to support the findings of this study are available from the corresponding author upon request.

## Supplementary Materials

Supplementary Materials Table S1: demographic details of the entire study population. Table S2: vital signs and EMS response of the entire study population. Table S3: odds ratios of in-hospital complications by type of injury, teaching status, profit status, and other vital parameters.
