# Supplementary material for: Effect of the Profit and Teaching Status of Hospitals on the Patterns and Outcomes of Abdominal Aorta and Inferior Vena Cava Injuries after Severe Abdominal Trauma
Source: Emerg Med Int. 2023 Aug 11;2023:5616007. doi: 10.1155/2023/5616007 (PMC10438973; doi:10.1155/2023/5616007)
Supplement: Supplementary Materials — Table S1: demographic details of the entire study population. Table S2: vital signs and EMS response of the entire study population. Table S3: odds ratios of in-hospital complications by type of injury, teaching status, profit status, and other vital parameters. [file 5616007.f1.docx]

**Table S1: Demographic details of the entire study population**

| \|  \| Total \| \| --- \| --- \| \|  \| (N = 1479) \| |
| --- | --- | --- | --- | --- |
| \| Age group \|  \| \| --- \| --- \| \| <16 \| 65 (4.5%) \| \| 16-20 \| 150 (10.3%) \| \| 21-44 \| 760 (52.1%) \| \| 45-64 \| 307 (21.0%) \| \| 65+ \| 178 (12.2%) \| \| Sex \|  \| \| Men \| 1147 (77.6%) \| \| Women \| 332 (22.4%) \| \| Race \|  \| \| White \| 734 (49.6%) \| \| Injury type \|  \| \| Blunt \| 766 (52.0%) \| \| Penetrating \| 705 (47.9%) \| \| Others \| 1 (0.1%) \| \| Mechanism \|  \| \| MVA \| 582 (39.7%) \| \| Firearms \| 638 (43.5%) \| \| Fall \| 84 (5.7%) \| \| Cut/Peirce \| 67 (4.6%) \| \| Others \| 94 (6.4%) \| \| Intent \|  \| \| Unintentional \| 772 (52.2%) \| \| Self-inflicted \| 55 (3.7%) \| \| Assault \| 604 (40.8%) \| \| Others \| 48 (3.2%) \| \| Verification Level \|  \| \| I - Level I Trauma Center \| 841 (75.2%) \| \| II - Level II Trauma Center \| 243 (21.7%) \| \| III - Level III Trauma Center \| 35 (3.1%) \| \| Beds \|  \| \| <= 200 \| 108 (7.3%) \| \| 201-400 \| 316 (21.4%) \| \| 401-600 \| 445 (30.1%) \| \| > 600 \| 610 (41.2%) \| |

MVA: Motor vehicle accidents

**Table S2: Vital signs and EMS response** **of the entire study population**

| \|  \| Total \| \| --- \| --- \| \|  \| (N = 1455) \| |
| --- | --- | --- | --- | --- |
| \| Length of stay, ICU (days) \|  \| \| --- \| --- \| \| Median (Q1, Q3) \| 5.0 (3.0, 11.0) \| \| Length of stay, hospital (days) \|  \| \| Median (Q1, Q3) \| 5.0 (1.0, 15.0) \| \| Time to EMS Response (mins) \|  \| \| Median (Q1, Q3) \| 7.0 (5.0, 13.0) \| \| Time from dispatch to hospital arrival (mins) \|  \| \| Median (Q1, Q3) \| 34.0 (25.0, 54.0) \| \| SBP \|  \| \| SBP>=90 \| 982 (67.5%) \| \| SBP<90 \| 473 (32.5%) \| \| GCS \|  \| \| GCS>8 \| 870 (59.8%) \| \| GCS<=8 \| 585 (40.2%) \| \| ISS Groups \|  \| \| 1-8 \| 1 (0.1%) \| \| 9-15 \| 65 (4.5%) \| \| 16-24 \| 357 (24.5%) \| \| 25+ \| 1032 (70.9%) \| \| AAI, IVCI, and both \|  \| \| AAI \| 639 (43.9%) \| \| IVCI \| 742 (51.0%) \| \| Both \| 74 (5.1%) \| \| Complications \| 495 (34.0%) \| \| Died Under Care \| 700 (48.1%) \| |

AAI: Abdominal aorta injury, GCS: Glasgow Coma Scale, ISS: Injury severity score, IVCI: Inferior vena cava injury, MVA: Motor vehicle accidents, SBP: Systolic blood pressure

# Table S3: The odds ratio of in-hospital complications by type of injury, teaching status, profit status, and other vital parameters

| **Variable** | OR[95% CI] | p-value |
| --- | --- | --- |
| Age | 0.999[0.993-1.006] | 0.824 |
| ISS | 1.007[0.999-1.015] | 0.105 |
| SBP | 1.011[0.782-1.308] | 0.934 |
| GCS | 0.729[0.564-0.942] | 0.016 |
| AAI/IVCI |  |  |
| AAI | 1 | . |
| IVCI | 1.659[1.31-2.101] | <.001 |
| Teaching status |  |  |
| CH | 1 | . |
| NTH | 0.594[.372-.949] | 0.029 |
| UH | 1.174[.913-1.509] | 0.212 |
| Profit status (NFPH) | 0.975[.664-1.431] | 0.895 |

AAI: Abdominal aorta injury, GCS: Glasgow Coma Scale, ISS: Injury severity score, IVCI: Inferior vena cava injury, MVA: Motor vehicle accidents, SBP: Systolic blood pressure
